# Oral health and emotional well-being in premenopausal and postmenopausal women: a cross-sectional cohort study

**DOI:** 10.1186/s12905-021-01480-5

**Published:** 2021-09-23

**Authors:** Nil Yakar, Asena Türedi, Gülnur Emingil, Çağdaş Şahin, Timur Köse, Angelika Silbereisen, Nagihan Bostanci

**Affiliations:** 1 Section of Oral Health and Periodontology, Division of Oral Diseases, Department of Dental Medicine, Karolinska Institutet, Alfred Nobels Allé 8, 14152 Huddinge, Sweden; 2 Department of Periodontology, Faculty of Dentistry, Ege University, İzmir, Turkey; 3 Department of Gynecology and Obstetrics, Faculty of Medicine, Ege University, İzmir, Turkey; 4 Department of Biostatistics and Medical Informatics, Faculty of Medicine, Ege University, Izmir, Turkey

**Keywords:** Depression, Menopause, Oral health, Osteoporosis, Periodontitis, Questionnaire

## Abstract

**Background:**

Menopause, the absence of ovarian sex steroids, is frequently accompanied by emotional and physiological changes in a woman´s body, as well as oral health changes. The present study aimed to evaluate the association between the periodontal health status and emotional and physical well-being among postmenopausal women (PMW) in comparison with regularly menstruating premenopausal women (RMPW).

**Methods:**

A total of 115 women (PMW, n = 56, mean age ± SD: 54 ± 5; RMPW, n = 59, mean age ± SD: 41 ± 4) received a comprehensive medical assessment and a full-mouth oral examination. All completed the Women’s Health Questionnaire (WHQ) to measure emotional and physical well-being. The corresponding bone mineral density (BMD) scores were obtained from participants´ medical records.

**Results:**

Tooth loss was significantly higher in PMW than RMPW after adjusting for age (3.88 ± 2.41 vs 2.14 ± 2.43, *p* < 0.05). No significant difference was found in the prevalence of periodontitis between the two groups (PMW: 39.2%, RMPW: 32.2%, *p* > 0.05). The prevalence of periodontitis was associated with fewer daily brushing sessions in PMW (*p* = 0.021). Based on the WHQ, both PMW and RMPW with periodontitis had higher ‘’depressed mood’’ scores compared to periodontally healthy women (*p* = 0.06 and *p* = 0.038, respectively). The women who reported fewer daily toothbrushing sessions found to have higher depressive mood scores (*p* = 0.043).

**Conclusions:**

Presence of periodontitis is associated with the emotional and physical well-being of women and reinforcement of oral healtcare is recommended at different stages of a woman’s life including menopause to reduce the risk for early tooth loss in women.

## Background

There are several stages in a woman’s life where changes in hormone levels make them more susceptible to oral diseases, including during menstruation, pregnancy, and menopause [1–3]. Menopause is a period in a woman’s lifetime, characterized by permanent cessation of menses, accompanied by major changes in sex hormone levels. The menopausal transition is frequently associated with concomitant physiological and psychological changes including vasomotor symptoms, sleep and mood disturbances [4]. Postmenopausal changes also affect intraoral tissues, as thinning of the oral mucosa, alterations of the oral flora, and decrease of alveolar bone mineral density [5–7].

Periodontitis is a chronic inflammatory disease of supporting tissues around teeth that may lead to tooth loss due to alveolar bone resorption. It occurs as a result of a dysbiotic subgingival flora in susceptible individuals [8, 9]. Although increased levels of ovarian hormones, as seen in puberty, pregnancy and menstruation, can result in an increase in gingival inflammation and microbial changes in dental plaque [1–3, 10], contrariwise, during menopause, a lack of hormones can also lead to poor periodontal health. Hormonal changes and decreased bone mineral density are proposed to increase predisposition towards alveolar bone loss in postmenopausal women [11]. Previous studies estimated menopause as a risk factor for periodontitis. However, controversial results were reported. So far, there is no consensus for an increased risk of periodontitis after menopause [12–14].

Individuals under emotional stress are more likely to develop periodontitis [15, 16]. Women are shown to be more prone to mental illnesses compared to men, representing a two-fold greater incidence [17]. Furthermore, the menopausal transition puts women at greater risk for depression [18]. Quality of life and psychological stress factors need to be investigated as confounders in oral health evaluations of postmenopausal women.

We hypothesized that certain psychosomatic characteristics could characterize women at greater risk of poor oral health before or during menopause. This study aimed to investigate the relationship between clinical periodontal health and physical and emotional symptoms extracted by Women’s Health Questionnaire (WHQ) scores, in postmenopausal women (PMW), in comparison with regularly menstruating premenopausal women (RMPW). We also evaluated systemic bone mineral density as a modifying factor of periodontitis in PMW.

## Methods

### Participants

This cross-sectional cohort study included 115 women with an age range of 35–65 years. The study was conducted between March and October 2019 at the Department of Periodontology, Faculty of Dentistry, Ege University, İzmir, Turkey in collaboration with the Department of Gynecology and Obstetrics, Faculty of Medicine, Ege University. The protocol was performed in accordance with the Declaration of Helsinki and approved by the Ege University Faculty of Medicine Ethics Committee for Clinical Research (18-10/61). All participants signed an informed consent and the study conforms to the STROBE guidelines for human observational studies.

The study population comprised of two main groups, postmenopausal women (PMW) and regularly menstruating premenopausal women (RMPW). The women in the PMW group were included if they were under 65 years of age and had a history of spontaneous amenorrhea within the last 12 months. The women in the RMPW group were included as participants if they were above the age of 35 years and without any irregularity in their menstrual cycles within the past 12 months. All women had at least 10 natural teeth without any prosthetic restoration. Women were excluded if they used antibiotics within the last 3 months, if they went through menopause after a medical attempt, or if they had ongoing cancer therapy or another acute disease or diabetes.

### Periodontal examinations

A total of 115 women (PMW: N = 56, RMPW: N = 59) who fulfilled the inclusion criteria were asked to complete standardized questionnaires to acquire demographic data, self-reported systemic health history, age (years) at the visit and at menopause, weight and height, lifestyle/oral care habits, frequency of dental visits, education, and marital status prior to periodontal examinations. The full-mouth periodontal examination was conducted by the trained and calibrated examiners (AT, GE, weighted kappa = 0.887 for probing depth (PD)). The following periodontal parameters were recorded from six surfaces per tooth: PD, gingival recession (GR), bleeding on probing (BOP), and plaque index (PI) [19]. PD was defined as the distance in millimeters from the gingival margin to the base of the gingival sulcus measured using a manual probe (Michigan 0 probe with Williams markings) in all the teeth present except for third molars. Similarly, clinical attachment level (CAL) was measured as the distance from the cementoenamel junction to the base of the pocket and recorded manually to the nearest millimeter marking on the probe.

**Fig. 1:**
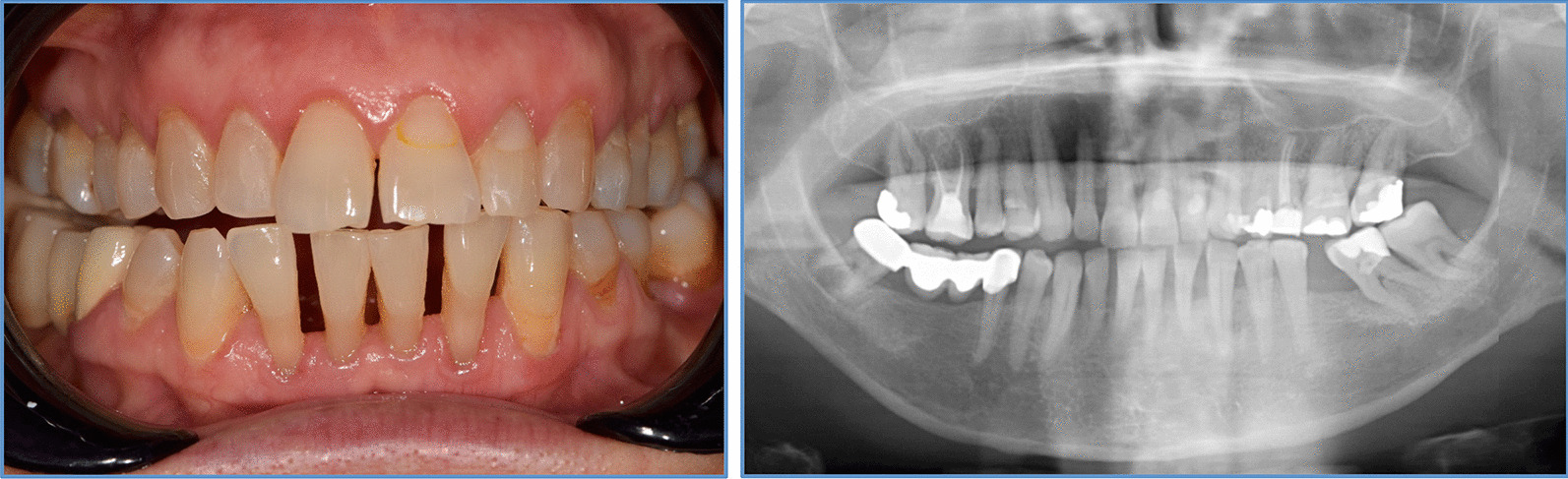
Clinical and radiographic images of a representative patient from the postmenopausal group with periodontitis diagnosis

Periodontal diagnosis was made according to the 2017 classification for periodontal and gingival diseases [20] under consideration of clinical presentation and patients’ medical history. Participants were categorized based on their periodontal status into periodontitis (Stage I/II-Moderate and Stage III/IV-Severe) or non-periodontitis (gingivitis and healthy). Individuals were diagnosed with periodontitis if interdental CAL was detectable at ≥ 2 non-adjacent teeth, or if buccal/oral CAL ≥ 3 mm with pocketing > 3 mm at ≥ 2 adjacent teeth was present. Periodontitis patients were grouped as ‘’severe periodontitis’’ (Stage III and IV), if they had a history of tooth loss due to periodontitis, CAL ≥ 5 mm and/or PD ≥ 6 mm and/or vertical bone loss ≥ 3 mm and/or furcation involvement grade 2 or 3 was present. Milder cases were recorded as ‘’moderate periodontitis’’ (Stage I and II). PD ≤ 3 mm and BOP ≥ 10% was recorded as ‘’gingivitis’’. Individuals with PD ≤ 3 mm and full mouth BOP < 10% were recorded as ‘’healthy’’. Figure 1 shows clinical and radiographic images from one representative within the postmenopausal group with clinically diagnosed periodontitis.

### The women’s health questionnaire

The Turkish version of the Women’s Health Questionnaire (WHQ) [21], attained from https://eprovide.mapi-trust.org and previously validated [22, 23], was directed to all participants. WHQ is a questionnaire which has been developed to assess a mid-aged woman’s short-term well-being in nine sections; depressed mood, somatic symptoms, vasomotor symptoms, anxiety/fears, sexual behavior, sleep problems, menstrual symptoms, memory/concentration, and attractiveness with a total of 37 questions. The sections ‘’menstrual symptoms’’ and ‘’sexual behavior’’ were excluded during evaluation due to unanswered questions [24]. The answers to the questionnaire were defined as ‘’Yes, definitely’’, ‘’Yes, sometimes’’, ‘’No, not much’’ and No, not at all’’. However, the calculation is based on binary scoring as 0 or 1, instead of a 4-point score. Every question took a score of 0 or 1. The sum of the scores in the related section was divided by the number of questions and each section was scored between 0–1. Scores approaching 1 indicated that the individual experienced symptoms related to a particular domain more prominently.

### Bone mineral density scores

As the PMW group consisted of participants who were referred to the Gynecology Clinics, the bone densitometry measurement results (femur and lumbar spine, recorded as either normal, osteopenic, or osteoporotic) for 39 of the women were available in the hospital’s electronic records and included in the assessment.

### Statistical analysis

Power analysis was performed using G*Power 3.1.9.2 version [25]. Fifty-three volunteers for each group were sufficient to demonstrate statistical significance between the two groups for 0.05 Type-I error and 80% power with a medium effect significant (d = 0.05). An extra 10% participants were included to overcome the potential information loss due to incomplete questionnaire data (59 participants in each group). Three participants from the PMW group were excluded due to incomplete questionnaires. Statistical analyses were conducted with the statistical software SPSS v. 19.0 (IBM, Somers, NY, USA). The Chi-Square test or Fisher’s exact test was used to investigate the association between categorized variables. After the Kolmogorov-Smirnov normality test, multiple group comparisons were performed with the Kruskal–Wallis test, if the data was not normally distributed. In cases of a significant result with the Kruskal–Wallis test, two-group comparisons were performed with Dunn Test with Bonferroni correction. For normally distributed numerical parameters, one-way ANOVA was performed for multiple group analysis. Two-group comparisons were performed with a t-test or Mann-Whitney-U test. The linear relationship between ordinal and numeric variables was analyzed with Spearman’s correlation analysis. Analysis of covariance (ANCOVA) was applied for the assessment of tooth loss difference between the two groups after adjusting for age.

## Results

### Demographics

The questionnaires collected self-reported information on demographics, overall health status, oral hygiene habits, and smoking. The participants’ age ranged from 35 to 65 years, the mean age was 54 ± 5 years in the PMW group and 41 ± 4 years in the RMPW group. The mean age of menopausal transition was 46 ± 4 years (Table 1).

**Table 1:** Characteristics of participants (N = 115)

Variables		PMW (n = 56) Mean ± SD or n (%)	RMPW (n = 59) Mean ± SD or n (%)	*p* value
Age (years)		54 ± 5	41 ± 4	< 0.0001*
Menopause age (years)		46 ± 4		
*Education*				
Primary		17 (30.4%)	6 (10.2%)	0.019^†^
Secondary		11 (19.6%)	19 (32.2%)	
High		28 (50%)	34 (57.6%)	
*Smoking*				
Yes		18 (32%)	18 (30.5%)	0.95^†^
No		38 (68%)	41 (69.5%)	
BMI		27.55 ± 5.41	25.61 ± 4	0.036*
Systemic disease prevalence		33(58.9%)	23(38.9%)	0.032^†^

*BMI* Body Mass Index

*PMW* post-menopausal women

*RMPW* regularly menstruating premenopausal women

*T-test for equality of means

^†^Pearson chi-square test

### Educational status and smoking

The education level was higher in the RMPW group (*p* = 0.019). There was no significant difference between the two groups regarding smoking status (*p* = 0.95) (Table 1).

### Systemic health and body mass index

Thirty-three patients in the PMW group (58.9%) and 23 patients in the RMPW group (38.9%) reported the presence of at least one systemic disease. The difference between the groups was significant (*p* = 0.032). In addition, BMI was significantly higher in PMW than RMPW (*p* = 0.036, mean ± SD 27.55 ± 5.41, 25.61 ± 4, respectively).

### Oral findings

#### Tooth number

The mean number of missing teeth was significantly higher in the PMW group (*p* < 0.0001). After co-variance analysis based on age, the difference remained significant (*p* = 0.048) (Table 2). Presence of periodontitis did not significantly affect the number of missing teeth (*p* = 0.88 for PMW, *p* = 0.16 for RMPW).

**Table 2:** Oral findings and oral care habits of participants

Characteristics	PMW group (N = 56) Mean ± SD or n (%)	RMPW group (N = 59) Mean ± SD or n (%)	*p*-value*
Number of missing teeth	3.88 ± 2.41	2.14 ± 2.43	< 0.0001, 0.048^†^
*Periodontal diagnoses*			
Healthy	2 (3.6%)	3 (5.1%)	0.42
Gingivitis	32 (57.1%)	37 (62.7%)	
Periodontitis	22 (39.3%)	19 (32.2%)	
Moderate	12 (21.4%)	8 (13.6)	0.73
Severe	10 (17.8%)	11 (17.5%)	
*Toothbrush frequency*			
< 1/day	4 (7.1%)	3 (5.1%)	0.623
1/day	19 (33.9%)	25 (42.4%)	
≥ 2/day	33 (58.9%)	31 (52.5%)	
*Dental visit frequency*			
Over a complaint	41 (71.2%)	42 (73.2%)	0.522
1/year	10 (13.6%)	8 (17.9%)	
2/year	5 (8.9%)	9 (15.3%)	

*PMW* post-menopausal women

*RMPW* regularly menstruating premenopausal women

*Pearson chi-square test

^†^After age adjustment

#### Periodontal measures

After full-mouth clinical examinations, 22 patients in the PMW group (39.3%) and 19 patients in the RMPW group (32.2%) were diagnosed with periodontitis (Table 2). There was no significant difference between PMW and RMPW regarding the prevelance of periodontitis (*p* = 0.42). Periodontitis patients compared to non-periodontitis patients displayed higher mean value for the duration after menopause (9.19 ± 6.05 years and 7.37 ± 8.69 years, respectively), and a younger menopause age (45.76 ± 4.17 years and 47.68 ± 3.96 years, respectively). However, differences were not significant (*p* = 0.88 and *p* = 0.091, respectively). Smoking prevelances (PMW periodontitis: 38,1% non-periodontitis: 32.4%; RMPW, periodontitis: 42.7%, non-periodontitis: 25%) and BMI were similar between periodontitis and non-periodontitis groups. (Smoking; PMW: *p* = 0.172, RMPW: *p* = 0.674) (BMI; PMW: *p* = 0.971, RMPW: *p* = 0.181). In the PMW group, the prevelaence of periodontitis was associated with significantly fewer daily brushing sessions (*p* = 0.021).

### Oral care habits

The majority of women (58% of PMW and 52% of RMPW) were reportedly brushing their teeth ≥ 2 times/day and 70.94% of women were visiting a dentist upon a complaint. The intergroup comparisons differed statistically for neither frequency of dentist visits nor daily toothbrushing (*p* = 0.52 and *p* = 0.62, respectively) (Table 2).

### The women’s health questionnaire outcomes

Mean scores of seven domains in the WHQ are summarized in Table 3. Scores of somatic symptoms, vasomotor symptoms, sleep problems and attractiveness were significantly higher in PMW compared to RMPW (*p* = 0.002, *p* < 0.0001, *p* = 0.005, and *p* = 0.001, respectively). However, scores for ‘’depressed mood’’, “memory/concentration” and “anxiety/fears” did not differ significantly between the two groups (*p* > 0.05).

**Table 3:** Questionnaire scores among study groups

Section	Score*		*p* value^†^
	PMW group (N = 56) Mean ± SD	RMPW group (N = 59) Mean ± SD	
Depressed mood	0.26 ± 0.21	0.21 ± 0.16	0.146
Somatic symptoms	0.46 ± 0.25	0.31 ± 0.23	0.002
Memory/Concentration	0.39 ± 0.35	0.33 ± 0.35	0.416
Vasomotor symptoms	0.45 ± 0.45	0.09 ± 0.21	< 0.0001
Anxiety/Fears	0.27 ± 0.26	0.23 ± 0.26	0.484
Sleep problems	0.47 ± 0.35	0.28 ± 0.33	0.005
Attractiveness	0.40 ± 0.41	0.16 ± 0.32	0.001

*PMW* post-menopausal women

*RMPW* regularly menstruating premenopausal women

*Extension of the scoring scale is between 0–1.

^†^T-test for equality of means

#### Relationship of periodontal diagnoses with depressive mood and sleep problems domains

**Fig. 2:**
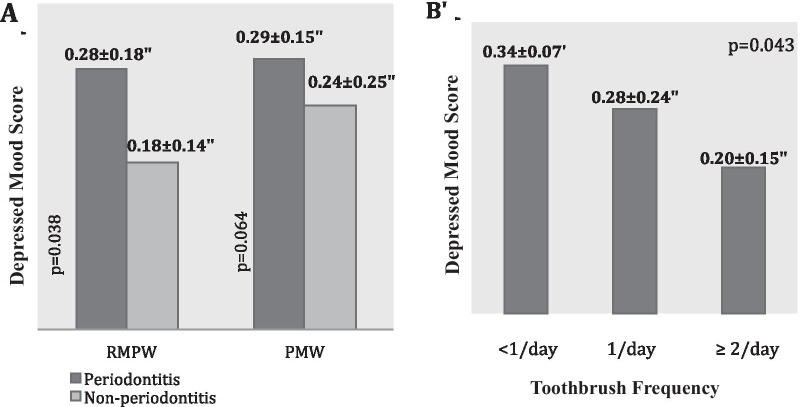
** A** Comparison of depressed mood scores (Mean ± SD) in regularly menstruating premenopausal women (RMPW) and post-menopausal women (PMW) with or without periodontitis. *P* values are given for Mann Whitney-U Test. **B** Daily toothbrush frequencies concerning depressed mood scores (Mean ± SD) in the entire cohort (RMPW and PMW together). *P*-value is given for Kruskal–Wallis Test

In the RMPW group, periodontitis patients had significantly higher ‘’sleep problems’’ (*p* = 0.043) and ‘’depressive mood’’ (*p* = 0.038) scores compared to patients without periodontitis. In PMW, participants with periodontitis had higher mean ‘’depressive mood’’ score compared to participants without periodontitis (0.29 ± 0.15 and 0.24 ± 0.25, respectively) (*p* = 0.06) (Fig. 2A). The women (PMW and RMPW) who reported fewer daily toothbrushing sessions found to have higher depressive mood scores (*p* = 0.043) (Fig. 2B).

### Bone mineral density diagnoses

In PMW, 39 patients’ electronic reports for osteoporosis were available categorized as ‘’normal’’, ‘’osteopenic’’ or ‘’osteoporotic’’. The diagnoses were based on the lumbar spine (L1-L4) and femur neck T score measurements, according to WHO criteria [26]. There was no significant difference for osteoporosis diagnoses between periodontitis and non-periodontitis groups (Lomber area, p = 0.454, femoral area, *p* = 0.328) (Table 4).

**Table 4:** Bone diagnoses and periodontitis in PMW

Diagnoses (N = 39)	Periodontitis (N = 14) (n,% within periodontitis)	Non-Periodontitis (N = 25) (n, % within non-periodontitis)	*p*-value*
*Lomber area*			0.454
Normal	6 (42.9%)	6 (24%)	
Osteopenic	5 (35.7%)	13 (52%)	
Osteoporotic	3 (21.4%)	6 (24%)	
*Femoral area*			0.328
Normal	5 (35.7%)	13 (52%)	
Osteopenic	9 (64.3%)	11 (44%)	
Osteoporotic	0 (0%)	1 (4%)	

*Pearson chi-square test

## Discussion

There are several stages in a woman’s life during which changes in hormone levels make them more susceptible to oral diseases, including menopause [1–3]. The present findings indicate that women after menopause experience more tooth loss, higher BMI and concomitant systemic diseases. Moreover, the presence of periodontitis is associated with higher depressive mood scores in women but not with osteoporotic changes.

An association between postmenopause and higher numbers of missing teeth has been supported by Alves et al. [13]. Yet, they described that there is a tendency for a more extensive tooth loss among postmenopausal women compared to the premenopausal controls. Nevertheless, this association did not remain significant after adjusting for the confounding factors. Reportedly, low bone mineral density [27, 28], the number of pregnancies [29] or hypertension [30] are among the risk factors related to tooth loss in women. Pan et al. suggested poor oral hygiene as a major contributing factor for tooth loss during menapose, rather than bone mineral density [31]. Hormone replacement therapy was reported to be associated with longer tooth retention [30, 32].

Although alterations in sex hormone levels affect periodontal tissues and lead to poorer periodontal health [1, 5, 33, 34], the present study did not identify significant differences regarding periodontal parameters between the two studied groups. There was also no association between osteoporosis scores and increased risk of periodontitis in postmenopausal women [35, 36]. While the majority of the studies reported a positive association between systemic bone mineral density and radiographic alveolar bone crest height [7, 12, 37–39], the association between osteoporosis and clinical parameters of periodontitis are inconclusive [37, 40, 41]. Supportively, LaMonte et al. found no positive correlation between menopause age or duration of postmenopausal life with periodontitis in more than a thousand postmenopausal women who had been followed up for 5 years [12]. This is further supported by earlier studies demonstrating that menopause may not pose a risk to the periodontium in postmenopausal women with good oral health [42].

The present findings may not be suprising, as the relationship between menopause and periodontitis is complex due to the number of factors involved. In the present study, in particular, mean age differences between the two groups were approximately 13 years. Nevertheless, the group of postmenopausal women had higher number of missing teeth after age adjustment. However, it is arguable that age, smoking, socio-economic factors as well as systemic conditions may play a role as confounders on the relationship between menopause and missing teeth [12]. Furthermore, tooth loss is of complex aetiology, reflecting cumulative conditions of oral health over time [43].

We also found significantly higher ‘’depressive mood’’ scores in both premenopausal and postmenopausal women who had periodontitis. In line with the present findings, an earlier study in Japanese females between 40 and 59 years of age showed that depressive tendencies during menopause are more common among women with oral health problems [44]. The WHQ has been widely used to evaluate the efficacy of medical interventions such as hormone replacement therapies or non-medical interventions (i.e. exercise) upon the quality of life scores [45]. Although oral health-related quality of life is a widely studied subject in the general population [46, 47] and also in postmenopausal women [48, 49], to the best of our knowledge no previous study used the WHQ to assess the link between the quality of life scores and periodontal health. Premenopausal women may also be susceptible to depressive mood at times of hormonal transitions [50]. There are several hypothesis to explain the association between depression and periodontal diseases and they share common risk factors such as poor oral hygiene, smoking and low socioeconomic status [15, 51]. The present findings indicated that the association between depressive mood and periodontitis might be mediated by oral hygiene habits as both pre/postmenopausal women reported less frequent tooth brushing. Earlier reports indicated that depressive mood is related to the negligence of oral health care [52]. Additionally, increased dental plaque levels may lead to an inflammatory breakdown of the periodontal tissues, and vice-versa, systemic inflammation induced by the presence of periodontitis may lead to depression [53]. Since both depression and periodontits are of chronic nature, further follow-up studies would be required in order to evaluate their progression patterns during menapouse.

## Conclusions

In conclusion, the present study demonstrated that there is an association between the number of missing teeth, poor emotional well-being, and menopause. Women with a history of periodontitis may experience concomitant emotional problems or women with depressive mood may be more susceptible to periodontitis. The WHQ is of great value for assessment of psychosocial effects of menopause in women with poor oral health. Nevertheless, the presented data due to its cross-sectional design may not be sufficient to understand the oral health trends during pre- and postmenopause. Additional multi-centered, larger follow-up studies are needed in order to confirm or exclude the role of menopause on poor oral health.

## Data Availability

The datasets used during the current study are available from the corresponding author on reasonable request.

## Abbreviations

PMW: Postmenopausal women
RMPW: Regularly menstruating premenopausal women
WHQ: Women’s health questionnaire
BMD: Bone mineral density
BMI: Body mass index
PD: Probing depth
GR: Gingival recession
BOP: Bleeding on probing
PI: Plaque index
CAL: Clinical attachment level
SD: Standard deviation
HRT: Hormone replacement therapy
